# Novel Stimulation Paradigms with Temporally-Varying Parameters to Reduce Synchronous Activity at the Onset of High Frequency Stimulation in Rat Hippocampus

**DOI:** 10.3389/fnins.2017.00563

**Published:** 2017-10-10

**Authors:** Ziyan Cai, Zhouyan Feng, Zheshan Guo, Wenjie Zhou, Zhaoxiang Wang, Xuefeng Wei

**Affiliations:** ^1^Ministry of Education Key Lab of Biomedical Engineering, College of Biomedical Engineering and Instrument Science, Zhejiang University, Hangzhou, China; ^2^Department of Biomedical Engineering, College of New Jersey, Ewing, NJ, United States

**Keywords:** high frequency stimulation, pulse stimulation, intensity transition, frequency transition, axonal conduction block, LabVIEW software

## Abstract

Deep brain stimulation (DBS) has shown wide applications for treating various disorders in the central nervous system by using high frequency stimulation (HFS) sequences of electrical pulses. However, upon the onset of HFS sequences, the narrow pulses could induce synchronous firing of action potentials among large populations of neurons and cause a transient phase of “onset response” that is different from the subsequent steady state. To investigate the transient onset phase, the antidromically-evoked population spikes (APS) were used as an electrophysiological marker to evaluate the synchronous neuronal reactions to axonal HFS in the hippocampal CA1 region of anesthetized rats. New stimulation paradigms with time-varying intensity and frequency were developed to suppress the “onset responses”. Results show that HFS paradigms with ramp-up intensity at the onset phase could suppress large APS potentials. In addition, an intensity ramp with a slower ramp-up rate or with a higher pulse frequency had greater suppression on APS amplitudes. Therefore, to reach a desired pulse intensity rapidly, a stimulation paradigm combining elevated frequency and ramp-up intensity was used to shorten the transition phase of initial HFS without evoking large APS potentials. The results of the study provide important clues for certain transient side effects of DBS and for development of new adaptive stimulation paradigms.

## Introduction

Electrical stimulation therapy has wide applications in both central and peripheral nervous systems for treating clinic patients. For example, deep brain stimulation (DBS) has been used to treat brain disorders, such as Parkinson's disease, essential tremor, epilepsy, addiction, and depression (Udupa and Chen, 2015; Wichmann and DeLong, 2016). Stimulation of peripheral nerves can be used to modulate motor and sensory nerve activity, such as to restore functional bladder control (Franke et al., 2014; McGee et al., 2015).

Most stimulation therapies utilize high-frequency stimulation (HFS), though the frequency ranges of HFS have different definitions. For brain stimulations, a pulse frequency >50 Hz is defined as HFS (Durand and Bikson, 2001). For example, regular DBS of subthalamic nucleus (STN) utilizes stimulation frequencies of 90 – 185 Hz as efficient frequencies for treating tremor in clinic (Rizzone et al., 2001; Kuncel et al., 2006). However, kilohertz frequencies have been used for stimulating peripheral nerves to induce conduction block for therapy (Franke et al., 2014; McGee et al., 2015).

Regardless of the frequency ranges of HFS, based on cybernetics, the responses of nervous systems to a prolonged HFS sequence may have a transient phase before establishment of a steady phase. In fact, undesirable transient phases (termed as “onset response”) have been reported at the beginning of kilohertz HFS on peripheral motor nerve fibers before an establishment of complete conduction block (i.e., steady phase). The onset response is a highly synchronous activation of a population of axons (so called a transitory volley) and can induce tetanus of targeted muscles thereby causing painful sensation (Bhadra and Kilgore, 2005).

However, to our knowledge, so called “onset response” has not been defined in DBS. Nevertheless, transient side effects (e.g., paresthesias) have been observed in clinical DBS that could be the evidence of HFS-induced “onset response” in STN stimulation (Kuncel et al., 2006). Additionally, electrographic potentials of synchronous firing of neurons (an analog of epileptiform activity) have been observed at the initial phases of HFS in other brain regions, such as hippocampus, in animal experiments (Jensen and Durand, 2009; Kim et al., 2012; Feng et al., 2013). Therefore, a transient phase characterized by highly synchronous activation could always appear at the HFS onsets regardless of central or peripheral nervous systems. The synchronous neuronal activation might not induce sensation and/or muscle activity directly and severely in DBS because of the specific stimulation region and stimulation paradigms used by the present DBS therapy. For example, for regular DBS persisting for hours, the effect of transient phases only lasting few seconds may be insignificant. However, it may be significant for intermittent or cycle stimulation paradigms used in adaptive or closed-loop stimulations. Frequent repetition of highly synchronous actions in neurons is a potential harm to brain. Therefore, in the present study, we tested novel HFS sequences with ramp-up intensity and elevated frequency to suppress the onset responses induced by axonal HFS in brain.

To investigate the onset responses of HFS in brain electrographically, we chose the hippocampal CA1 region of rat brain and recorded the antidromically-evoked population spikes (APS) during axonal HFS. Because of the dense packing of the neuron's cell bodies in the region, the APS potentials are used as a marker to evaluate the number of neurons that fire synchronously and to reveal the temporal dynamics of HFS responses. Also, the lamellar structures of axonal fibers, soma layer and dendrite layer of the hippocampal region provide separate and clear locations for the axonal stimulation and for the neuronal recording (Kloosterman et al., 2001).

In addition, regular stimulators only permit delivery of pulse stimuli at fixed intensity and frequency at each stimulation session. To obtain various stimulation paradigms with time-varying intensity and time-varying frequency, we designed a LabVIEW program to control the output waveforms of a commercial stimulator by using a data acquisition card. The output pulse trains with desired paradigms were then used in rat experiments. The results of the study provide novel stimulation paradigms that could have potential applications for extending DBS treatments in future.

## Materials and methods

### Configuration and design of the stimulation system

**Figure 1:**
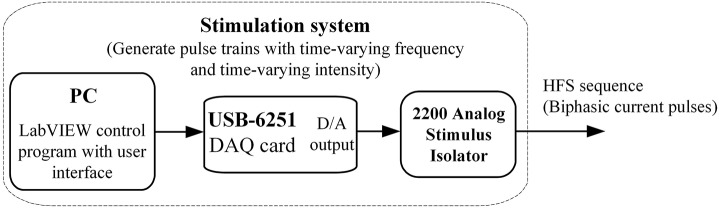
Schematic diagram of the stimulation system.

The stimulation system was designed by using a PC, a USB-6251 data acquisition card (DAQ card, National Instruments), and a 2200 analog stimulus isolator (A-M Systems Inc.). A custom-made LabVIEW program was run to control a D/A converter in the DAQ card to generate desired sequences of voltage pulses. The voltage pulses were then converted into current pulses by the 2200 stimulus isolator to be finally delivered into a rat brain through a stimulation electrode (Figure 1).

The core of the LabVIEW program is the generation of biphasic pulse sequences with time-varying parameters. Two sequences of monophasic pulses (0.1 ms width) were multiplied into a biphasic unity sequence. Then, the unity sequence was multiplied by linearly increasing coefficients to obtain a pulse sequence with ramp-up intensity. In addition, a sequence with attenuating-frequency (from 400 to 100 Hz) was designed by linearly increasing inter-pulse intervals (IPI) in the pulse sequence. Therefore, the corresponding change of pulse frequency follows a curve with a sharp initial decline gradually flatting down to the final desired frequency. Such a design can shorten the phase of higher frequencies and smooth the connection between the onset transient phase of HFS with varying parameters and the subsequent period of HFS with constant parameters. The final HFS sequence was built by sequentially combining several pieces of pulse sequences with required parameters to form a compound stimulation sequence.

To verify the outputs of the stimulation system, we connected the current pulses generated from the 2200 stimulus isolator to a 10 kΩ resistor mimicking the resistance of brain tissue and measured the intensities (0.01 – 0.5 mA), frequencies (50 – 500 Hz) and pulse width (0.1 ms). The errors of intensity and frequency were all smaller than 1% and the error of pulse width was 2.7%.

In addition, although an analog arbitrary signal generator can be used to output arbitrary waveforms, it lacks a friendly interface to program the outputs on-line. The stimulation system developed here with the virtual instrument technology of LabVIEW can meet the demands easily.

### *In vivo* animal experiment, stimulation, and recording

All procedures involving animal care and experiments conformed to the Guide for the Care and Use of Laboratory Animals (China Ministry of Health). The surgical procedure and data collection methods were similar to previous reports (Feng et al., 2013, 2014). Briefly, adult Sprague Dawley rats (250 – 400 g, 10) were anesthetized with urethane (1.25 g/kg, i.p.) and placed in a stereotaxic apparatus. Electrodes were inserted into the hippocampal CA1 region through partially opened skull.

A concentric bipolar stainless-steel stimulation electrode (Model CBBSC75, FHC), was positioned in the alveus, the efferent fiber tracts of hippocampal CA1 region, for antidromically exciting the CA1 pyramidal cells. The inner polar of the electrode delivered biphasic pulses with cathodic phase first while the outer polar was connected as a return electrode. A recording electrode (16-channel array, Model Poly2, NeuroNexus Technology) was positioned in the CA1 pyramidal layer, upstream of the stimulating site, to collect the antidromically evoked responses. To ensure correct positions of the electrodes, we placed a second stimulation electrode in the Schaffer collaterals, the afferent fiber tracts of CA1 region for orthodromically exciting the CA1 neurons. According to the patterns of orthodromically- and antidromically-evoked potentials as well as unit spike signals appeared serially in the 16 channel recording array, the electrode sites can be assessed (Kloosterman et al., 2001).

The electrophysiological signals collected by the recording electrode array were amplified by a 16-channel extracellular amplifier (Model 3600, A-M System Inc.), then sampled by a PowerLab 16/35 data acquisition system (ADInstruments Inc.) at a sampling rate of 20 kHz/channel, and stored into a hard disk for offline analysis.

**Figure 2:**
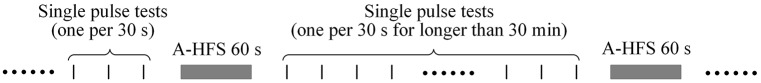
Schematic timeline of stimulation paradigms. The idle intervals between sequential A-HFS sequences were longer than 30 min to ensure recovery from the previous stimulation. Single pulse tests (one per 30 s) were run in the intervals to monitor the changes of evoked APS.

Two to four different stimulation paradigms were run in each animal. Each paradigm included a pair of test sequence and its control sequence. Thus, multiple antidromical HFS (A-HFS) sequences were run in each animal. The order of sequences was random. The idle intervals between sequential A-HFS sequences were longer than 30 min to ensure full recovery from previous stimulation (Figure 2). Single pulse tests (one per 30 s) were run in the idle intervals to monitor the changes of evoked APS. For all of the 57 A-HFS sequences, the APS amplitudes recovered over 90% in 2.5 min following the termination of A-HFS.

The stimulation pulses were all biphasic current pulse with cathodic phase first and 0.1 ms pulse width per phase. The intensity of current pulses was 0.2, 0.3, or 0.4 mA. A single pulse at this intensity range was able to induce a large APS with an amplitude of ~75% maximum APS in the input-output curves. These intensities were used as desired intensities for all the single pulse tests, all the stimulation sequences with constant intensity, and all the subsequence phases of stimulation following the initial transition phases with ramp-up intensity in a rat. The range of ramp-up intensity was 0.02 – 0.2, 0.03 – 0.3, or 0.04 – 0.4 mA with the desired intensity as the upper limit (the final intensity) and a tenth of the desired intensity as the lower limit that would not induce any obvious APS.

Statistical data were represented as mean ± standard deviation. One-way ANOVA with post-hoc Bonferroni's test was used to judge the statistical significance of the differences among three data groups. Paired *t-*test was used to judge the differences between a test stimulation sequence and its control sequence from the same animals.

## Results

### Neuronal responses to HFS with time-varying intensity in rat hippocampus

**Figure 3:**
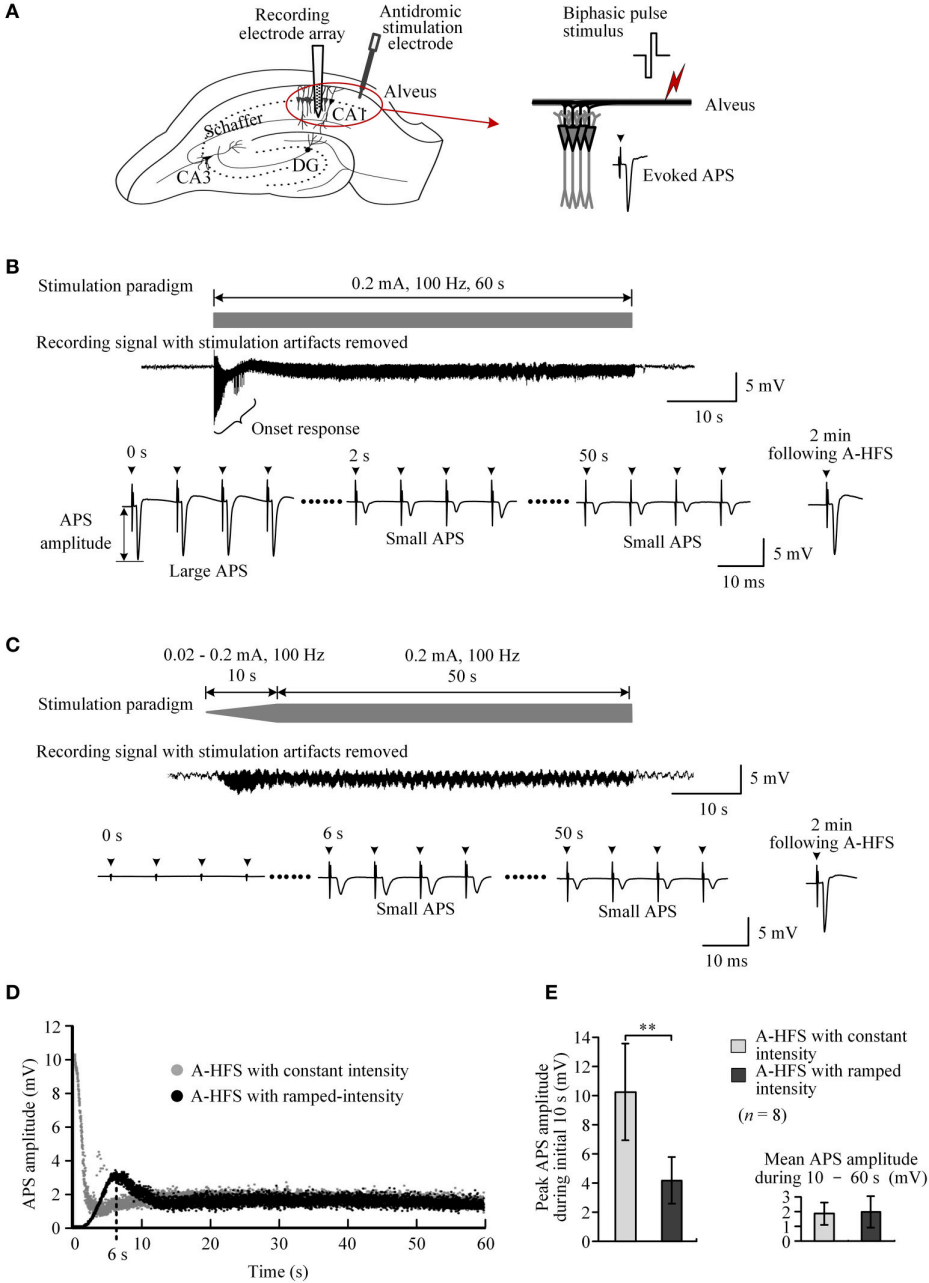
Comparison of neuronal activity induced by A-HFS sequences with constant intensity and with ramp-up intensity. **(A)** Schematic diagrams of the locations of recording electrode array and stimulation electrode in the hippocampal CA1 region, waveforms of biphasic pulse stimulus and antidromically-evoked population spike (APS). **(B)** Top: diagram of 100 Hz A-HFS sequence with a constant intensity of 0.2 mA and a duration of 60 s. Middle: an example of evoked APS (with stimulation artifacts removed) during the A-HFS. Bottom: Expanded APS waveforms at 0, 2, and 50 s from the onset of A-HFS, and the recovered APS at 2 min following A-HFS. **(C)** Top: diagram of 100 Hz A-HFS with ramp-up intensity (0.02–0.2 mA) for the beginning 10 s phase before a constant intensity (0.2 mA) for the remaining 50 s. Middle: Evoked APS during A-HFS. Bottom: Expanded APS waveforms at the onset (0 s), at 6 and 50 s of A-HFS together with the recovered APS at 2 min following A-HFS. Arrows above the expanded waveforms in **(B,C)** denote the artifacts of A-HFS. **(D)** Scatter diagrams of the amplitudes of APS evoked by each pulse during the two A-HFS paradigms. **(E)** Statistical comparisons between the two paradigms for the peak APS amplitudes at the beginning 10 s (left) and the mean APS amplitudes during the late 10 – 60 s of A-HFS (right). ^**^P < 0.01, paired *t*-test, *n* = 8.

Extracellular stimulation of alveus fibers may activate axons and antidromically induce action potentials in the cell bodies of CA1 neurons (Figure 3A). During a 60 s A-HFS sequence of 100 Hz with a constant intensity (0.2 mA), APS potentials followed each pulse, but their amplitudes changed dynamically. At the onset of A-HFS, every pulse evoked a large APS with an amplitude (>10 mV) similar to the APS evoked by a single pulse stimulation in baseline tests. However, the APS amplitudes decreased to 1 ~ 2 mV in 2 s and remained small until the end of stimulation. Two minutes after the end of A-HFS, the APS evoked by a test pulse recovered (Figure 3B). The decrease of APS amplitudes during the A-HFS may be caused by failures of axons to generate an action potential following each pulse of the prolonged A-HFS sequence (Jensen and Durand, 2009; Feng et al., 2014).

To prevent large APS at the onset of HFS, we designed and tested an A-HFS sequence with a ramp-up intensity (0.02 – 0.2 mA) at the initial 10 s phase before the remaining 50 s stimulation with constant intensity (0.2 mA). The starting intensity (0.02 mA) was too small to induce any obvious APS at the onset of HFS. With the increase of pulse intensity, the APS gradually appeared and increased in amplitude (Figure 3C). However, no large APS appeared throughout the entire stimulation period even when the intensity reached the maximum value (0.2 mA), the same as the control A-HFS with constant intensity. The peak APS (only ~3 mV) appeared at ~6 s when the intensity increased to ~0.13 mA (Figure 3D). During the late ~40 s period with an intensity of 0.2 mA, the APS amplitudes overlapped with the values evoked by the control stimulation with a constant intensity (0.2 mA) from the very beginning (Figure 3D).

Statistical data from eight experiments using 100 Hz pulse sequences showed that the mean peak APS amplitude at the initial 10 s phase with ramp-up intensity was only 41.0 ± 7.7% of the values at the same A-HFS phase but with constant intensity. The difference between the peak APS amplitudes of the two paradigms was significant (*P* < 0.01, paired *t*-test, *n* = 8). The mean APS amplitudes during 10 – 60 s of the two paradigms were similar (*P* = 0.48, paired *t*-test, *n* = 8; Figure 3E).

**Figure 4:**
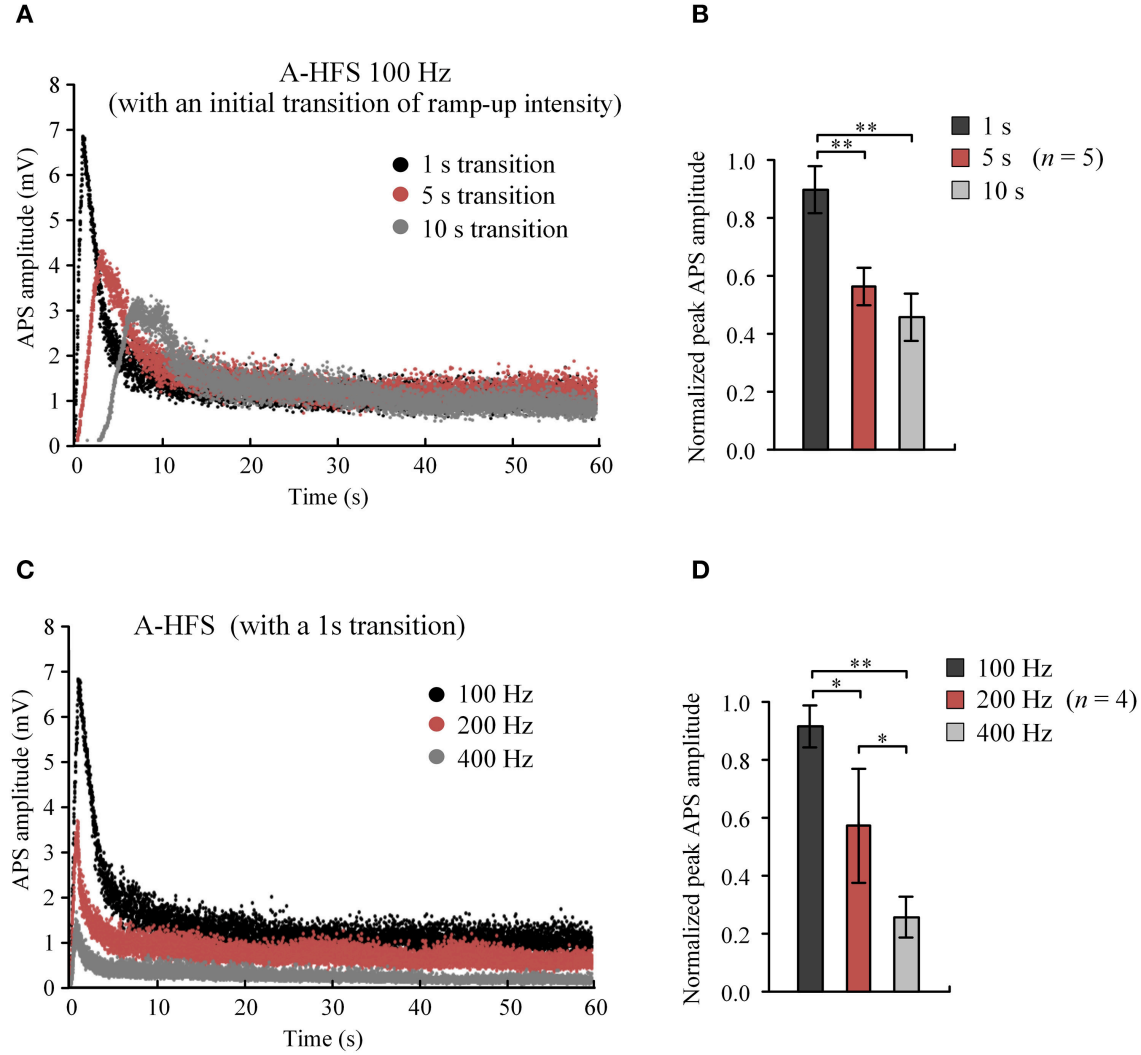
Comparisons of APS amplitudes evoked by A-HFS sequences with different durations of ramped-intensity transition and with different pulse frequencies. **(A)** Examples of APS amplitudes during three A-HFS sequences (100 Hz, 60 s) with different transition phases of 1, 5, and 10 s ramped-intensity (0.03–0.3 mA) in the beginning phase and a constant intensity (0.3 mA) for the remaining A-HFS period. **(B)** Comparisons of the normalized peak APS amplitudes among the three paradigms shown in **(A)**. ^**^P < 0.01, ANOVA with *post-hoc* Bonferroni's test, *n* = 5. **(C)** Examples of APS amplitudes during three stimulation paradigms with different frequencies of 100, 200, and 400 Hz, with only 1-s transition phase of ramped-intensity (0.03–0.3 mA) in the beginning. **(D)** Comparisons of the normalized peak APS amplitudes among the three paradigms shown in **(C)**. ^*^P < 0.05, ^**^P < 0.01, ANOVA with *post-hoc* Bonferroni's test, *n* = 4.

These results indicate that A-HFS with ramp-up intensity starting from small stimulation could avoid generating large APS events at the onset of HFS. However, to suppress large APS with 100 Hz A-HFS, a transition phase of 10 s was required for the stimulation intensity to increase to a desired strength. Shortening transition phases resulted in less suppression of APS amplitudes (Figure 4A). A 1 s short transition only decreased the peak APS to ~90% and was significantly larger than 5 s (~50%) and 10 s (~40%) transitions (Figure 4B). Nevertheless, elevating the frequency to 400 Hz in the 1 s short transition suppressed APS significantly >100 and 200 Hz (Figures 4C,D). Therefore, we next tested a stimulation paradigm with a higher pulse frequency at the beginning of A-HFS to shorten the transition phase of ramp-up intensity. A greater stimulation intensity generates a larger action area (i.e., volume of tissue activated). Thus, a rapid increase of the ramp-up intensity could allow the action of stimulation reaching a desired area immediately following the onset of stimulation.

### Neuronal responses to HFS combining varying intensity with varying frequency

**Figure 5:**
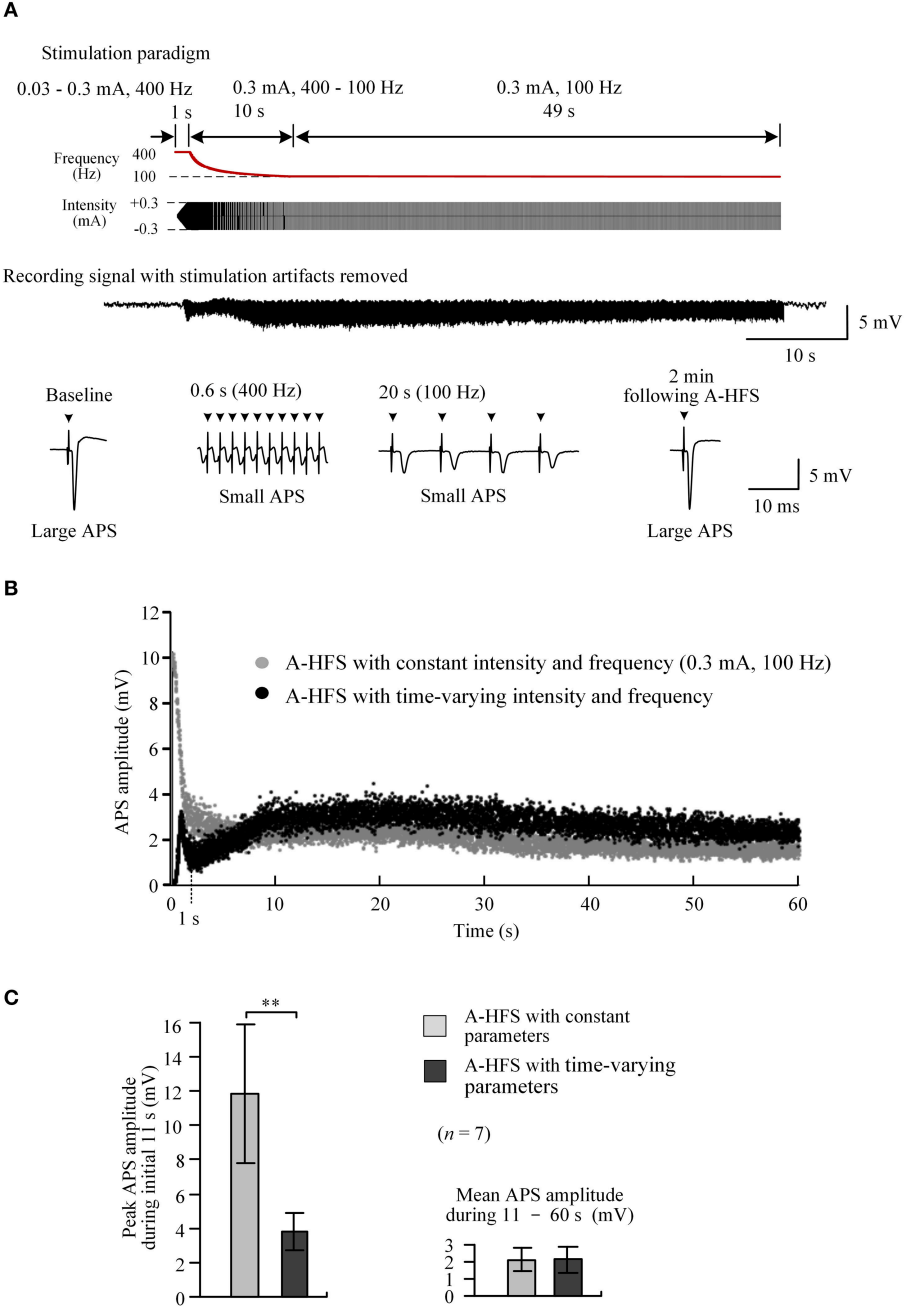
Neuronal activity induced by A-HFS with varying intensity and varying frequency. **(A)** Top two rows: schematic diagrams of 60 s A-HFS sequence comprised by a ramped-intensity (0.03–0.3 mA) phase at 400 Hz for 1 s, an attenuating-frequency (400–100 Hz) phase at 0.3 mA for 10 s, followed by a constant-intensity (0.3 mA) and constant-frequency (100 Hz) phase for the remaining 49 s. Bottom two rows: an example of evoked APS during the entire A-HFS and expanded APS waveforms at baseline, 0.6 and 20 s from the onset of A-HFS, and the recovered APS at 2 min following A-HFS. **(B)** Scatter diagrams of the amplitudes of APS evoked by each pulse during A-HFS with varying parameters and with constant parameters. **(C)** Statistical comparisons between the two paradigms for the peak APS amplitudes at the beginning 11 s (left) and the mean APS amplitudes during the late 10 – 60 s of A-HFS (right). ^**^P < 0.01, paired *t*-test, *n* = 7.

To rapidly reach desired stimulation intensity while avoiding large APS events at the onset of A-HFS, we tested a 60 s pulse sequence composed of three sub-sequences sequentially: a 1 s sub-sequence with an elevated frequency of 400 Hz and ramped intensity from 0.03 to 0.3 mA (linear incline); a 10 s sub-sequence with a constant intensity of 0.3 mA and attenuating frequency from 400 Hz to desired 100 Hz (hyperbolic decline); and a remaining 49 s period with a constant intensity of 0.3 mA and a constant frequency of 100 Hz (Figure 5A, top).

At a high frequency of 400 Hz, during the first 1 s phase when the stimulation intensity increased to a desired strength, the peak APS amplitude was only 3.2 mV (10.3 mV for corresponding control A-HFS). Then, during the following 10 s phase with attenuating frequency from 400 Hz to desired 100 Hz, the APS amplitudes slightly increased from a trough (Figure 5B). Finally, during the late 49 s period with constant 0.3 mA and 100 Hz, the APS amplitudes maintained at a small level and were similar to the corresponding APS evoked by a regular A-HFS sequence with constant parameters all the time (Figure 5B).

Statistical analysis showed that during the initial 11 s transition phase, the peak APS amplitude evoked by A-HFS with the time-varying paradigm was 33.1 ± 5.0% of the values at the same A-HFS phase but with a constant intensity. The difference between the peak APS amplitudes of the two paradigms was significant (*P* < 0.01, paired *t*-test, *n* = 7). The mean APS amplitudes during 11 – 60 s of the two paradigms were similar (*P* = 0.87, paired *t*-test, *n* = 7; Figure 5C).

## Discussion

In the present study, we applied new HFS paradigms with time-varying intensity and time-varying frequency to the alveus fiber of rat hippocampus *in vivo*. The stimulation paradigms decreased large antidromically-evoked population spikes at the onset of HFS. The neuronal responses and possible underlying mechanisms are discussed below.

### Plausible mechanisms of the neuronal response to time-varying stimulation of axons

In extracellular stimulation, a single electrical pulse or a brief sequence of pulses with suprathreshold intensity can activate a bundle of axonal fibers to synchronously generate action potentials propagating antidromically to cell bodies upstream. Consequently, a large population of neurons fire synchronously resulting in a large APS potential, just as that was recorded at the onset of the control A-HFS sequence with constant stimulation parameters (Figure 3B). However, the APS amplitudes declined rapidly with the stimulation continuing. Because antidromic activation involves only axons and cell bodies (Figure 3A), failures in the two elements of neurons should be responsible for the decline. Actually, previous studies have shown that prolonged axonal HFS with a frequency over 100 Hz may generate partial conduction block in axons and cause the axons to fail to respond to every pulse of HFS (Chomiak and Hu, 2007; Jensen and Durand, 2009; Zheng et al., 2011; Kim et al., 2012; Feng et al., 2013; Rosenbaum et al., 2014). Therefore, HFS-induced axonal failures could be the possible mechanism underlying the APS declines in the control A-HFS sequences.

The mechanism could also explain the disappearance of large APS in time-varying sequences. For a stimulation subsequence with ramp-up intensity, the intensity of initial pulses was too small to induce any obvious APS. By increasing pulse intensity, the APS gradually appeared and increased, indicating that a growing number of axons were activated with the enlargement of stimulation area by a greater pulse intensity. However, the APS did not grow to as large as the one evoked by a single pulse stimulation even though the pulse intensity ramped up to the same large value (Figures 3C,D). Additionally, the peak APS was smaller for a stimulation subsequence with a slower ramp-up intensity (Figures 4A,B) or with a higher pulse frequency (Figures 4C,D). Possibly, during the period with increasing intensity, the earlier activated axons could have entered a state of conduction failure earlier. Therefore, at a specific moment, each pulse could only generate action potentials in a small portion of the axons thereby keeping the APS amplitude at a low level. In addition, a slower rate of ramp-up intensity could allow enough time for the axons to enter failure states. A higher pulse frequency could force the axons to generate failures rapidly (Feng et al., 2013, 2014). Therefore, large APS events could be avoided by lowering the ramp rate of intensity or by utilizing a higher pulse frequency.

To reach a desired action area (volume of tissue activated) quickly at the onset of HFS, a large pulse intensity should be applied early. A paradigm combining a 1st transition phase of ramp-up intensity with 400 Hz elevated frequency and a 2nd transition phase of attenuating frequency from 400 Hz to desired 100 Hz could enable the simulation to reach the desired action area in 1 s while simultaneously avoiding large APS (Figure 5). The similar APS potentials evoked in the subsequent A-HFS following the initial transition periods, together with the quick recovery of APS after the end of whole A-HFS, indicate that no long lasting effects were caused by 400 Hz stimulation. Additionally, previous clinic investigations have shown that stable efficacy may remain from 90 Hz frequency up to a high frequency of 2,000 Hz (Limousin et al., 1995). Therefore, the 400 Hz frequency stimulation could be harmless except that it requires more power consumption at the short transition period.

These results suggest that large synchronous activity evoked at the onset of HFS could be suppressed by stimulation paradigms with time-varying parameters through a possible mechanism of HFS-induced axonal failures. To our knowledge, the present study is the first report on utilizing transitional HFS parameters to obtain a mild onset response in brain stimulation, although similar paradigms have been applied to peripheral nerve stimulation to minimize the onset responses (Bhadra and Kilgore, 2005; Miles et al., 2007; Bhadra et al., 2009; Gerges et al., 2010).

### Implication and limitation

Epileptiform activity, characterized by highly synchronous firing of neurons, has potential harm to brain tissue. Extensive experimental studies have demonstrated the epileptiform activity in electrophysiological recordings at the onset of axonal HFS in hippocampal region due to the dense neurons in the project area of activated axons (Jensen and Durand, 2009; Kim et al., 2012; Feng et al., 2013). Because axons are ubiquitous in brain and are easier to be excited by electrical pulses than other elements of neurons (Ranck, 1975), the effects of axonal HFS together with the “onset responses” could exist in HFS of any other brain regions. The phenomena might be difficult to detect because of lacks of large synchronous potentials due to a divergent distribution of innervated neurons. Nevertheless, they could be a potential cause for certain transient side effects observed in clinic DBS (Kuncel et al., 2006). The present study provides new insights for investigating the mechanisms of side effects and for developing novel stimulation paradigms of DBS.

Transient onset responses may be inconsequential to regular DBS persisting hours long. However, for adaptive or closed-loop DBS, cycle paradigms of HFS with minutes or shorter durations are often used, and the periodical appearance of onset responses at each initial phase of HFS could be consequential. Previous studies have shown that stimulations switched on and off for 1 – 60 s intervals decreased the beneficial effects of DBS in patients with Parkinson's disease, as compared to constant stimulation (Khandhar et al., 2005). Temporally cycling high-frequency DBS is less effective than regular high-frequency DBS at relieving tremor (Kuncel et al., 2012). The mechanisms underlying the decline of efficacy are not clear. The transient onset responses described in the present study could be one of the causes. HFS paradigms without inducing onset responses may provide an important clue for the development of adaptive stimulation that requires stimulator being turned on and off frequently based on the dynamic states of neuronal system (Hosain et al., 2014).

The present study was run on urethane anesthetized rats. Urethane is a widely used anesthetic in neurophysiological experiments. It has no severe influences on neuronal activity except for an elevation of the threshold of action potential firing (Sceniak and Maciver, 2006). In addition, any effects of urethane should have been minimized because the results used here were comparisons between the relative effects of different stimulation paradigms based on a same anesthetized state.

## Ethics statement

This study was carried out in accordance with the recommendations of Guide for the Care and Use of Laboratory Animals by China Ministry of Health. The protocol was approved by the Institutional Animal Care and Use Committee, Zhejiang University, Hangzhou.

## Author contributions

ZF, ZC, and XW designed the experiments and/or interpreted the data. ZC, ZG, ZW, and WZ performed the experiments and analyzed data. ZC developed the stimulation system. ZC and ZF drafted the manuscript. ZF and XW revised it critically for important intellectual content. All authors approved the final version of the manuscript to be published and agreed to be accountable for all aspects of the manuscript.

### Conflict of interest statement

The authors declare that the research was conducted in the absence of any commercial or financial relationships that could be construed as a potential conflict of interest.
